# Age-Related Differences in Senescence Marker Expression during Cutaneous Wound Healing

**DOI:** 10.1093/geroni/igaf122.1849

**Published:** 2025-12-31

**Authors:** Maria Shvedova, Chenyu Chu, Daniel Roh

**Affiliations:** Boston University, Boston, Massachusetts, United States; Boston University, Boston, Massachusetts, United States; Boston University, Boston, Massachusetts, United States

## Abstract

Impaired wound healing in older patients represents a significant and escalating clinical and economic challenge. Transient upregulation of cellular senescence signaling during wound healing is an important physiological mechanism that facilitates tissue repair. It is essential to differentiate senescent cells that accumulate with aging from the temporary upregulation of senescence signaling during acute wound healing. In this study, we compared cutaneous wound healing rates in 2-month-old (roughly corresponds to the age of 18 years in humans) and 24-month-old (roughly corresponds to the age of 70 years in humans) C57Bl/6 wild type male mice and examined differences in senescence marker expression. 24-months-old mice exhibited delayed wound healing and significantly reduced senescence marker expression, with a lack of p16, p21, and SA-β-gal upregulation, as well as altered inflammatory marker expression. Single-cell sequencing of wound tissue on day 6 after wounding - the peak of p16 expression - revealed that 91% (in 2-months-old mice) and 93% (in 24-months-old mice) of p16-positive cells were fibroblasts with an anti-inflammatory, extracellular matrix-producing phenotype. Notably, 24-month-old mice had 50% fewer p16-positive cells at this stage of wound healing. Given the essential role of fibroblasts in extracellular matrix deposition and tissue remodeling, these findings suggest that senescent fibroblasts may influence wound healing in a complex manner, with transient senescence playing a crucial role and aging impairing this physiological response. In conclusion, delayed wound healing in old mice is associated with decreased senescence signaling, highlighting a potential therapeutic target for improving wound repair in aging.

